# Alcohol Use Disorder and Schizophrenia or Schizoaffective Disorder

**DOI:** 10.35946/arcr.v40.1.06

**Published:** 2019-12-20

**Authors:** Luke Archibald, Mary F. Brunette, Diana J. Wallin, Alan I. Green

**Affiliations:** Luke Archibald, M.D., is an assistant professor in the Department of Psychiatry, Geisel School of Medicine at Dartmouth, Hanover, New Hampshire; Mary F. Brunette, M.D., is an associate professor in the Department of Psychiatry, Geisel School of Medicine at Dartmouth, Hanover, New Hampshire; Diana J. Wallin, Ph.D., is a postdoctoral fellow in the Department of Psychiatry, Geisel School of Medicine at Dartmouth, Hanover, New Hampshire; Alan I. Green, M.D., is the Raymond Sobel Professor of Psychiatry, a professor in the Department of Molecular and Systems Biology, and the chair of the Department of Psychiatry, Geisel School of Medicine at Dartmouth, as well as the director, Dartmouth Clinical and Translational Science Institute, Dartmouth College, Hanover, New Hampshire

**Keywords:** addiction, alcohol, pharmacotherapy, schizoaffective disorder, schizophrenia

## Abstract

Schizophrenia and schizoaffective disorder are schizophrenia spectrum disorders that cause significant disability. Among individuals who have schizophrenia or schizoaffective disorder, alcohol use disorder (AUD) is common, and it contributes to worse outcomes than for those who do not have co-occurring substance use disorder. Common neurobiological mechanisms, including dysfunction in brain reward circuitry, may explain the high rates of co-occurrence of schizophrenia and AUD or other substance use disorders. Optimal treatment combines pharmacologic intervention and other therapeutic modalities to address both the psychotic disorder and AUD. Further research on the etiology of these co-occurring disorders and on treatment of affected individuals is needed.

## Introduction

Schizophrenia and schizoaffective disorder are heterogeneous psychotic disorders that often cause significant disability, with symptoms that include delusions, hallucinations, disorganization, and cognitive impairment.^1^ In schizoaffective disorder, the psychotic symptoms are present, along with mood episodes of depression or mania.^2^ People with these schizophrenia spectrum disorders have high rates of co-occurring substance use disorder, including alcohol use disorder (AUD). This article provides an updated review of the epidemiology, neurobiologic basis of co-occurrence, assessment, and treatment of people with co-occurring AUD and schizophrenia or schizoaffective disorder.

## Epidemiology

The lifetime prevalence of schizophrenia is estimated to be about 1%.^1^ The lifetime prevalence of schizoaffective disorder is unknown, given changes in diagnostic criteria and challenges in differentiating this disorder from other diagnoses, but it is believed to be less common than schizophrenia, with regional estimates between 0.3% and 1.1%.^2^,^3^

Individuals with these psychotic disorders have three times the risk of heavy alcohol use relative to the general population.^4^,^5^ One meta-analysis of individuals with schizophrenia found a lifetime prevalence of AUD of 24.3%.^6^ One American study reported that 36.4% of 404 participants had experienced AUD before their first episode of psychosis.^7^ In both the general U.S. population and among people with schizophrenia, AUD is associated with male gender and Caucasian race.^7^ For individuals who have schizophrenia, AUD is associated with depression, suicidality, medication nonadherence, chronic physical problems, homelessness, aggression, violence, incarceration, and high rates of hospitalization.^7^–^10^

## Basis of Co-Occurrence

The genetic risk for schizophrenia has been fairly well-established. Heritability is estimated to be 80% to 85% for schizophrenia.^11^ Studies of twins have been a way to isolate genetic risk from environmental risk. The concordance rate, the likelihood that a second twin will receive a diagnosis of schizophrenia after the first twin, has been estimated at 41% to 65% for monozygotic and 0% to 28% for dizygotic twins.^11^ In addition, multiple genetic determinants of risk for schizophrenia (especially within neural systems) may contribute to the risk for both psychosis and addiction. For disorders such as schizophrenia that stem from variation at multiple genetic loci, the various risk alleles can be summed together to determine a polygenic risk score. Strong associations between substance use disorder, including AUD, and the polygenic risk score for schizophrenia indicate that shared genetic liability may contribute to the co-occurrence of these disorders.^12^

Several polymorphisms (genetic variations) of the brain-derived neurotrophic factor (BDNF) protein correlate with co-occurring schizophrenia and alcohol dependence but not with alcohol dependence alone, suggesting that these polymorphisms may contribute to a specific vulnerability to these co-occurring disorders.^13^ Recently, a large genome-wide association study of individuals with alcohol dependence (diagnosed using the fourth edition of the *Diagnostic and Statistical Manual of Mental Disorders*) revealed 17 traits, including schizophrenia, that had significant genetic correlations to alcohol dependence.^14^ These studies support the notion that certain genetic factors can lead to an increased risk for developing co-occurring schizophrenia and AUD.

Several theories have emerged to explain the high prevalence of co-occurring schizophrenia and substance use disorder.^8^,^15^ Rosenthal first proposed the diathesis-stress model in 1970 to describe the combined interaction of a neurobiological vulnerability with an environmental vulnerability that leads to the development of schizophrenia.^16^ This theory is also called the “two-hit” model. For the development of schizophrenia and AUD, for example, the two hits could be a genetic risk for schizophrenia combined with alcohol drinking during adolescence. Although alcohol use in adolescence predicts future co-occurring mental health disorders and substance use disorder, adolescent exposure to alcohol was not found to be associated with the age of onset of psychosis.^17^ A variant of the two-hit model is the cumulative risk factor hypothesis, which posits that among people with schizophrenia, the increased risk for developing substance use disorder stems from the added risks of poor cognitive development, poor social functioning, effects of poverty, and poor social environments.^1^

Another theory explaining the high rate of substance use disorder among individuals who have schizophrenia is the self-medication hypothesis, which suggests that people use substances to find relief from symptoms or in an effort to decrease side effects that arise from antipsychotic treatments.^18^ Although clinically plausible, this theory has not been supported by research. Studies indicate that negative symptoms are not necessarily elevated in individuals with schizophrenia and substance use disorder, and that among young people who experienced first-episode psychosis, substance use disorder often developed before the use of medications.^7^,^19^

In 2018, Khokhar and colleagues reviewed the unifying hypothesis that the co-occurrence of schizophrenia and substance use disorder may relate to a dysregulation of the mesocorticolimbic reward system in the brain.^15^ Sometimes called the primary addiction hypothesis^20^ or reward deficiency syndrome,^21^ this circuit-level dysregulation has been studied using functional magnetic resonance imaging (fMRI). People with co-occurring schizophrenia and nicotine dependence have been shown to have reductions in resting-state connectivity between the insula and the anterior cingulate cortex, and people with co-occurring schizophrenia and cannabis use disorder have been shown to have a hypoconnectivity between the nucleus accumbens and frontal cortical regions.^22^,^23^ Moreover, studies using task-based fMRI have reported dysfunction in the ventral striatum.^24^

The neurodevelopmental theory of schizophrenia suggests that an early insult in brain development may lead to onset of symptoms of schizophrenia in late adolescence or early adulthood.^25^ This theory led to the development of a putative animal model of schizophrenia—the neonatal ventral hippocampal lesion (NVHL) model. In this model, rats receive small, bilateral, hippocampal lesions at the end of the first week of life, and in adulthood they display many of the memory and social deficits associated with schizophrenia.^26^ This line of research is also promising for co-occurring substance use disorder, since NVHL rats consume more substances than their control group counterparts, and, after access to alcohol during adolescence, they drink more alcohol as adults.^27^ Thus, the NVHL rat may be a promising model for studying changes in the reward circuits of the brain among individuals who have schizophrenia and AUD, and for identifying potential therapeutic targets for those who have schizophrenia and co-occurring substance use disorder.

Although models of co-occurring AUD or substance use disorder and schizophrenia or schizoaffective disorder continue to evolve, understanding the basis of the co-occurrence may inform treatment approaches, especially pharmacologic treatment for the co-occurring disorders. Moreover, regardless of the model, it appears that AUD or substance use disorder and schizophrenia or schizoaffective disorder are linked. Thus, treatment for such co-occurring disorders must address both the psychotic symptoms and the alcohol or other substance misuse.

## Assessment, Treatment, and Prognosis

Given the high rates of co-occurring AUD among individuals with schizophrenia or schizoaffective disorder, as well as the clear evidence that such use can worsen the course of the psychotic disorder, diagnostic assessment for any individual presenting with symptoms of psychosis should include screening for alcohol and other substance use. In emergency departments, consideration of possible substance-induced psychosis is important. In one study, 18.9% of those with a diagnosis of substance-induced psychosis had alcohol as the primary substance.^28^ In 39.6% of cases, alcohol was used with cocaine or cannabis.

Because alcohol can precipitate psychotic symptoms during acute intoxication, withdrawal, or chronic use, obtaining a detailed history and creating a timeline of periods of psychotic symptoms and substance use can help clinicians differentiate between substance-induced psychosis and a primary psychotic disorder. Individuals with psychotic disorders may not be able or willing to provide these details of their history, particularly during periods of symptom exacerbation. Therefore, collecting information from collateral sources, such as family members, is often necessary. For initial evaluations and assessments of treatment response, laboratory examinations, such as testing for ethyl glucuronide, can provide useful evidence of recent alcohol use.^29^

Treatment for substance-induced psychosis focuses on acute management, often with reduced stimulation, in a supportive, abstinent environment, and sometimes with short-term antipsychotic treatment. Once an individual becomes abstinent and withdrawal has resolved, psychotic symptoms also usually resolve, but 25% of cases may persist, resulting in diagnoses of schizophrenia spectrum disorders.^30^ By contrast, treatment for individuals who have primary psychotic disorders with co-occurring AUD usually requires long-term antipsychotic medication and psychosocial interventions, in addition to other interventions for AUD noted in this section. Moreover, for individuals with co-occurring AUD and psychotic disorders, both disorders should be treated simultaneously. Thus, comprehensive treatment—combining medication with behavioral and psychosocial interventions—is appropriate.

### Pharmacologic treatment

This section reviews the evidence for the efficacy of medications used to treat AUD in individuals who have co-occurring schizophrenia or schizoaffective disorder. In addition, this section includes a review of the effects of antipsychotic medications on alcohol intake among individuals who have these co-occurring disorders.

#### AUD medication implications for schizophrenia or schizoaffective disorder

Several studies have examined the safety and efficacy of medications (i.e., naltrexone, disulfiram, and acamprosate) used to treat AUD in individuals with co-occurring schizophrenia and AUD.^31^ In a small, randomized controlled trial of patients with schizophrenia and AUD, those treated with naltrexone reported significantly fewer drinking days, fewer heavy-drinking days, and less craving, as compared to those receiving placebo.^32^ In a small, open-label study of naltrexone administered to individuals with schizophrenia spectrum disorders, investigators found improvements in various measures of alcohol intake, as well as in psychotic symptoms.^33^

Another study of patients with serious mental illnesses, including schizophrenia, compared naltrexone and disulfiram individually and in combination with placebo.^34^ In this study, participants who received active medication had better alcohol use outcomes than those who received placebo. However, the majority of participants with a psychotic spectrum disorder had a diagnosis of bipolar disorder, limiting the potential applicability for individuals who have schizophrenia or schizoaffective disorder.

No known studies have assessed extended-release naltrexone for AUD in a population that includes individuals with psychotic disorders. However, given the cognitive and executive dysfunction associated with schizophrenia, this formulation (injectable with slow release and gradual absorption over 4 weeks) of naltrexone may have potential benefits for increasing medication adherence.

A small, randomized controlled trial examined the use of acamprosate for individuals with psychotic disorders.^35^ In that study, all participants reduced drinking, and there was no difference between acamprosate and placebo in increasing the number of consecutive days of abstinence.

Theoretically, disulfiram has a risk of worsening psychosis in predisposed individuals because of its action of inhibiting dopamine beta-hydroxylase, but this phenomenon appears to be rare in clinical practice.^36^ Other than the study that compared naltrexone, disulfiram, or a combination of naltrexone and disulfiram, no known randomized controlled trials have examined disulfiram among individuals with psychotic disorders. However, in a chart review of 33 patients treated with disulfiram who had a diagnosis of alcoholism and also had severe mental illness, 64% experienced remission of the alcoholism for at least 1 year during a 3-year follow-up period.^37^

Few studies have examined use of other medications for off-label treatment of AUD in individuals with schizophrenia spectrum disorders. For example, the effects of topiramate on alcohol outcomes have not been studied in this population, although it has been used to potentially control weight in people with schizophrenia.^38^ There is some evidence that the mood stabilizer valproic acid may reduce alcohol consumption in a population that has dual diagnoses, which may have relevance for treating individuals with schizoaffective disorder. Specifically, in a randomized controlled trial of valproic acid versus placebo, in addition to treatment as usual, individuals with bipolar I disorder and alcohol dependence demonstrated a significantly smaller proportion of heavy-drinking days and a trend toward fewer drinks per heavy-drinking day.^39^ However, no known trials have examined valproic acid in a population of individuals with schizophrenia and AUD.

Varenicline, which has been approved by the U.S. Food and Drug Administration for the treatment of nicotine use disorder, has been shown to decrease alcohol consumption among participants with AUD.^40^ However, the only study of this medication in patients with schizophrenia and AUD reported poor tolerability.^41^ Benzodiazepines, although useful for treating alcohol withdrawal and as adjunctive agents for acute manic episodes, are not effective for treatment of AUD and are associated with worse outcomes, including risk of overdose when combined with alcohol.

In summary, although few studies have examined the effects of medications (i.e., naltrexone, disulfiram, and acamprosate) that treat AUD among individuals with psychotic disorders, evidence of the safety and potential benefit is sufficient to encourage increased use in this population (see Table 1).

#### Schizophrenia or schizoaffective disorder medication implications for AUD

The choice of medication for treating psychotic or affective symptoms in people with psychotic disorders may have implications for alcohol consumption. First-generation antipsychotic medications do not appear to decrease alcohol use and actually may increase substance use and craving in people with schizophrenia and co-occurring substance use disorder.^42^ Long-acting injectable formulations of second-generation antipsychotics, as well as clozapine, a novel second-generation antipsychotic, may be preferred.

A hypothetical framework has been delineated that supports the use of clozapine to ameliorate the brain circuit dysfunction experienced by people with schizophrenia and substance use disorder and is related to clozapine’s weak dopamine D_2_ receptor blockade coupled with its noradrenergic effects.^21^,^43^ Some evidence supports the superiority of clozapine for people who have schizophrenia and AUD.^43^ In a naturalistic, prospective study that followed patients with schizophrenia or schizoaffective disorder and co-occurring substance use disorder, a larger proportion of individuals receiving clozapine, versus those taking another atypical antipsychotic, achieved remission from AUD.^44^ During the following year, the participants who were in remission and were being treated with clozapine had lower rates of relapse to substance use than participants who were treated with other antipsychotics.^45^ Additional evidence from chart reviews and retrospective studies (see Table 2) favors the use of clozapine over other atypical antipsychotics.^46^

Long-acting injectable formulations of antipsychotics may help improve adherence or clarify when nonadherence is present, which may be particularly relevant in a dual-diagnosis population. In a randomized controlled trial comparing oral and long-acting injectable risperidone treatment for individuals with schizophrenia and AUD, heavy drinking worsened over time for those in the oral risperidone group compared to those treated with the long-acting injectable formulation.^47^ However, another study comparing long-acting injectable versus oral risperidone did not find differences in alcohol use outcomes between the two groups.^48^

Lastly, a randomized, open-label, review board–blinded study comparing once-monthly paliperidone palmitate to daily oral antipsychotics examined real-world outcomes for participants, a majority of whom had a diagnosis of co-occurring substance use disorder.^49^ This trial demonstrated the superiority of long-acting injectable paliperidone, including for the primary outcome of time to first treatment failure.

Other second-generation antipsychotics that do not have potent dopamine D_2_ blockade may have theoretical benefit over typical antipsychotic medications, although evidence in prospective controlled trials is limited. It has been postulated that the unique mechanism of action of aripiprazole (a partial agonist at dopamine D_2_ and 5-HT_1A_ receptors and an antagonist at 5-HT_2A_ receptors) may have beneficial effects for alcohol use.^50^ Uncontrolled trials provide support for use of aripiprazole among people who have co-occurring schizophrenia and cocaine or tobacco use disorder but not co-occurring schizophrenia and AUD.^51^ Quetiapine, which weakly blocks dopamine D_2_ receptors, has support from small, open-label trials that showed reductions in alcohol use.^52^ However, no randomized controlled trials of these medications examine alcohol outcomes in people with co-occurring schizophrenia and AUD.

### Psychotherapeutic and psychosocial interventions

Chronic psychotic illness is often accompanied by cognitive deficits and diminished executive functioning, which may be worsened by the effects of alcohol in those who have co-occurring AUD. Therefore, integrated and tailored care for both the psychotic disorder and AUD can improve access to care, deliver consistent messages about treatment and recovery, provide interventions that support attempts to reduce substance use, and manage behavioral health conditions.^53^

Group therapy using cognitive behavioral therapy, motivational enhancement therapy, or contingency management has a role in treating AUD and co-occurring schizophrenia.^54^,^55^ Considerations for this particular population include using active and ongoing motivation enhancement approaches and modifying cognitive behavioral therapy to account for cognitive, interpersonal, and motivational deficits that commonly occur among people with schizophrenia.^29^

Contingency management involves agreed on, immediate, tangible rewards to reinforce positive behaviors, such as treatment attendance or abstinence that has been verified by biologic measures. Such a management strategy for alcohol abstinence has been shown to be effective for people who have schizophrenia or other serious mental illness and who also have AUD. For example, one study demonstrated that participants who received contingency management intervention were 3.1 times more likely than participants from the control group to have a negative result on a urine test for the alcohol biomarker ethyl glucuronide.^56^ Also, these participants were more likely to attain 1.5 weeks of additional alcohol abstinence during a 12-week trial as compared to participants in the control group.

More intensive interventions, including assertive community treatment (ACT) and residential programs, may benefit individuals with co-occurring schizophrenia and AUD. ACT is the most widely tested model of community care for people with severe mental illness. ACT consists of an interdisciplinary team (i.e., the psychiatrist, social workers, nurses, occupational therapists, and peer support) with a low participant-to-staff ratio. This team provides a range of comprehensive services, including community outreach, 24-hour availability for emergency communication, and integrated pharmacotherapy and behavioral treatments for substance use disorder. For people with dual disorders, faithful implementation of and adherence to the ACT model is associated with superior outcomes in substance use, including significantly fewer days of alcohol and drug use.^57^ Residential programs that integrate treatment for mental health and substance use disorders can be effective and may be especially indicated for individuals who are homeless or have had suboptimal response to other interventions.^53^

Alcoholics Anonymous is underused among individuals with co-occurring AUD and psychotic disorders, although this population has unique considerations. People who have psychotic disorders benefit from the education and support they receive by attending and processing 12-step meetings, but people who have acute psychosis may not be able to tolerate these meetings.^58^ Dual Recovery Anonymous (Double Trouble or Double Trouble in Recovery) is a 12-step program tailored for individuals with co-occurring mental illness and substance use disorder. Evidence shows higher rates of abstinence, better adherence to psychiatric medication, and improved personal functioning for people who attended dual-focused groups as compared to those who attended Alcoholics Anonymous.^59^

## Future Research Directions

Additional research related to co-occurring AUD and schizophrenia or schizoaffective disorder is needed. Environmental factors, including substance use, that contribute to the risk of developing schizophrenia continue to be investigated. Prospective longitudinal markers of neurobiological function in adolescence before onset of psychotic symptoms and alcohol consumption could further elucidate the etiology of these disorders. Moreover, further development of evidence-based interventions to address alcohol and other substance use in adolescents before and during first-episode psychosis is required. Lastly, additional investigations into the efficacy of various treatment modalities are necessary, particularly because individuals with co-occurring disorders often are excluded from clinical trials.

## Figures and Tables

**Table 1:** Studies of Pharmacologic Interventions for AUD Among Individuals Who Have Schizophrenia Spectrum Disorders

Medication	Participants and Design	Results
Naltrexone^32^	Individuals (*N* = 31) with schizophrenia and co-occurring alcohol abuse or dependence* were treated with naltrexone (50 mg) or placebo, in addition to neuroleptic medication, for a 12-week, randomized controlled trial.	Participants treated with naltrexone, compared to those who received placebo, had significantly fewer drinking days and fewer heavy-drinking days (defined as more than five drinks), and they reported less craving.
Naltrexone and Disulfiram^34^	Individuals (*N* = 254) with alcohol dependence* and heterogeneous psychiatric disorders were treated with disulfiram and naltrexone alone and in combination. They also received intensive psychosocial treatment during the 12-week, randomized controlled trial.	Individuals with a psychotic spectrum disorder who received an active medication had better alcohol use outcomes when compared with those who received placebo. Neither disulfiram nor naltrexone nor the combination had a clear advantage.
Disulfiram^37^	In this retrospective review, individuals (*N* = 33) with alcohol abuse or dependence* and severe mental illness had been treated with disulfiram.	At a 3-year follow-up, 64% of individuals experienced remission of alcohol abuse or dependence* for at least 1 year.
Acamprosate^35^	Individuals (*N* = 23) with a diagnosis of alcohol dependence* and co-occurring schizophrenia, schizoaffective disorder, or nonspecified psychosis received acamprosate or placebo in a randomized controlled trial.	All participants reduced drinking. Acamprosate was not superior to placebo in increasing consecutive days of abstinence. Participants who received acamprosate reported significantly fewer obsessive thoughts of drinking than those who received placebo.
Valproic Acid^39^	Individuals (*N* = 59) with bipolar I disorder and alcohol dependence* received either valproate or placebo in a randomized controlled trial. All participants received treatment as usual (which included lithium).	The group that received valproate had a significantly smaller proportion of heavy-drinking days and a trend toward fewer drinks per heavy-drinking day when compared to the group that received placebo.
Varenicline^41^	Individuals (*N* = 55) with schizophrenia or schizoaffective disorder and concurrent alcohol and nicotine dependence* received varenicline or placebo in a pilot, 8-week, randomized controlled trial.	Because of safety concerns or loss to follow-up, only 10 participants started the study. Five received varenicline and five received placebo. Adverse gastrointestinal effects such as severe abdominal pain limited study completion to four participants.

* Study used the classifications of alcohol abuse and alcohol dependence as defined in the fourth edition of the *Diagnostic and Statistical Manual of Mental Disorders*.

**Table 2:** Studies of Antipsychotic Medications Among Individuals With Substance Use Disorder

Medication	Participants and Design	Results
Clozapine^44^,^45^	Patients (*N* = 151) with schizophrenia or schizoaffective disorder and co-occurring substance use disorder, of whom 36 were prescribed clozapine, were followed in a prospective study. The same patients were followed over the next year.	A larger proportion of participants who received clozapine, versus those taking a different atypical antipsychotic, achieved remission from AUD (79% vs. 34%). Participants in remission who had been treated with clozapine had lower rates of relapse to substance use (8%) than those treated with other antipsychotics (40%).
Clozapine and Risperidone^46^	In this retrospective review, patients with schizophrenia or schizoaffective disorder and co-occurring alcohol or cannabis use had been treated with clozapine or risperidone.	Abstinence rates were significantly higher for participants treated with clozapine than for those treated with risperidone (54% vs. 13%, *p* = .05).
Long-Acting Injectable Risperidone^47^	Individuals (*N* = 95) with schizophrenia and AUD received 6 months of risperidone either by long-acting injection or by mouth in a randomized controlled trial.	In the group that received risperidone by mouth, heavy drinking significantly worsened over time (*p* = .024). A slight difference between groups was shown for change in the number of heavy-drinking days per week, with the long-acting injection group showing a small decrease (*p* = .054). The long-acting injection group had significantly fewer drinking days per week than the by-mouth group (*p* = .035).
Long-Acting Injectable Risperidone^48^	Patients with schizophrenia who were unstable were treated with long-acting injectable or by-mouth risperidone in a randomized controlled trial. The length of time to psychiatric rehospitalization, as well as other clinical outcomes such as substance misuse, were examined.	Patients treated with long-acting injectable risperidone and those treated with by-mouth risperidone had no difference in alcohol use outcomes.
Long-Acting Injectable Paliperidone Palmitate^49^	Participants (*N* = 450) received either once-monthly paliperidone palmitate or daily oral antipsychotics in a 15-month, open-label, review board–blinded study. A majority of participants had a diagnosis of schizophrenia with co-occurring substance use disorder. Real-world outcomes were examined.	Results demonstrated superiority of long-acting injectable paliperidone, including for the outcome of time to first treatment failure.
